# Mucosal Genes Expression in Inflammatory Bowel Disease Patients: New Insights

**DOI:** 10.3390/ph16020324

**Published:** 2023-02-20

**Authors:** Sumaiah J. Alarfaj, Sally Abdallah Mostafa, Walaa A. Negm, Thanaa A. El-Masry, Marwa Kamal, Mohamed Elsaeed, Ahmed Mohamed El Nakib

**Affiliations:** 1 Department of Pharmacy Practice, College of Pharmacy, Princess Nourah Bint Abdulrahman University, P.O. Box 84428, Riyadh 11671, Saudi Arabia; 2 Department of Medical Biochemistry and Molecular Biology, Faculty of Medicine, Mansoura University, Mansoura 35511, Egypt; 3 Department of Pharmacognosy, Faculty of Pharmacy, Tanta University, Tanta 31527, Egypt; 4 Department of Pharmacology and Toxicology, Faculty of Pharmacy, Tanta University, Tanta 31527, Egypt; 5 Department of Clinical Pharmacy, Faculty of Pharmacy, Fayoum University, Fayoum 63514, Egypt; 6 Department of General Surgery, Faculty of Medicine, Mansoura University, Mansoura 35511, Egypt; 7 Department of Tropical Medicine, Faculty of Medicine, Mansoura University, Mansoura 35511, Egypt

**Keywords:** 5-ASA, anti-TNF, gene expression, Crohn’s disease, ulcerative colitis

## Abstract

Individual differences in IBD illness severity, behavior, progression, and therapy response are evident. Since a break in the intestinal epithelial barrier causes IBD to begin, mucosal gene expression in IBD is crucial. Due to its high sensitivity and dynamic nature, molecular analysis of biomarkers in intestinal biopsies is feasible and provides a reliable means of evaluating localized inflammation. The goal of this investigation was to discover alterations in gene expression in the inflamed mucosa of IBD patients undergoing treatment with 5-amino salicylic acid (5ASA) (N = 39) or anti-TNF drugs (N = 22). The mucosal expression of numerous IBD-related genes was evaluated using qPCR. We discovered that the levels of the proteins Lipocalin-2 (LCN2), Nitric Oxide Synthase 2 (NOS2), Mucin 2 (MUC2), Mucin 5AC (MUC5AC), and Trefoil factor 1 (TFF1), which are overexpressed in untreated IBD patients compared to non-IBD subjects, are decreased by both therapy regimens. On the other hand, anti-TNF medicine helped the levels of ABCB1 and E-cadherin return to normal in IBD patients who were not receiving treatment.

## 1. Introduction

Inflammatory bowel disease is a long-term inflammatory condition of the gut that can present clinically as Crohn’s disease (CD), ulcerative colitis (UC), or IBD undefined (IBD-U) [1,2,3].

The intensity, behavior, progression, and response to therapy for IBD disease show significant individual diversity [2,4,5,6]. Both clinical factors and molecular signatures have been linked to many elements of disease progression. Previous studies dealing with molecular investigations have been demonstrated and are used for clinical practice and therapeutic decision-making in IBD patients [7,8,9].

In the past 20 years, numerous innovative therapeutic agents targeting different immune pathways have also been developed, in addition to tumor necrosis factor (anti-TNF) inhibitors [10]. Taking these medications has improved the long-term results for both CD and UC, despite a significant increase in the expense burden on many health care systems [11].

The current therapy goals for IBD now include steroid-free remission, endoscopic remission, and lowering surgery rates [12]. Although the primary therapeutic purpose was to alleviate patient symptoms, in addition to the fact that over 30% of IBD patients do not respond to treatment, among those who do, the response vanishes in 23–46% of instances after a year of medication use [13]. In order to improve the overall IBD disease management, a treatment plan based on molecular perturbations and the detection of predictors of nonresponse can be chosen [14].

In IBD patients, mucosal gene expression is particularly significant because a breach in the intestinal epithelium barrier is thought to be the source of the disease’s development. Due to their high sensitivity and dynamic nature, various molecular biomarkers in intestinal biopsies are feasible and offer a reliable approach to evaluating localized inflammation.

Mucosal genes play an essential role in mucosal integrity and play an important role in the pathogenesis of IBD. Genes within several IBD-associated loci indicate a position for barrier integrity in disease predisposition [15].

Another difficulty in controlling IBD disease is monitoring the pathophysiological mechanisms behind the chronic inflammatory process and the effects of treatment. Studies on molecular mucosal profiling are scarce in this area [15].

This study aimed to find differences in the gene expression between the mucosa of IBD patients receiving 5-aminosalicylic acid (5ASA) or biological therapies in the form of anti-TNF treatment and IBD patients receiving no medication or control subjects without IBD.

## 2. Results

### 2.1. Histological Changes

**Figure 1:**
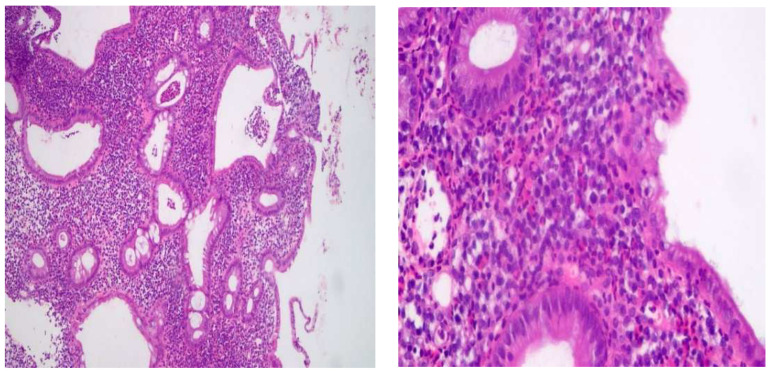
Moderate diffuse active colitis consistent with IBD (ulcerative colitis).

**Figure 2:**
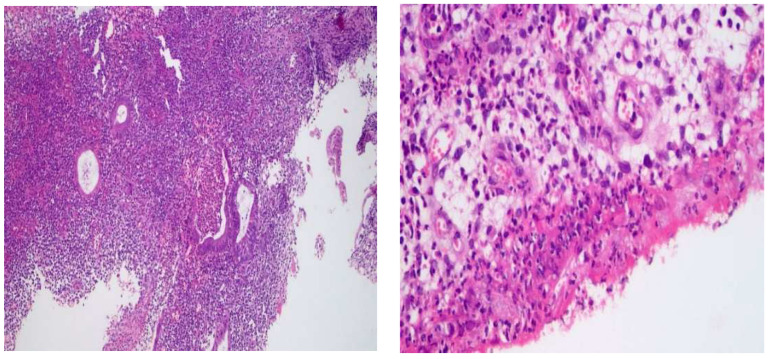
Severe diffuse active colitis consistent with IBD (ulcerative colitis).

**Figure 3:**
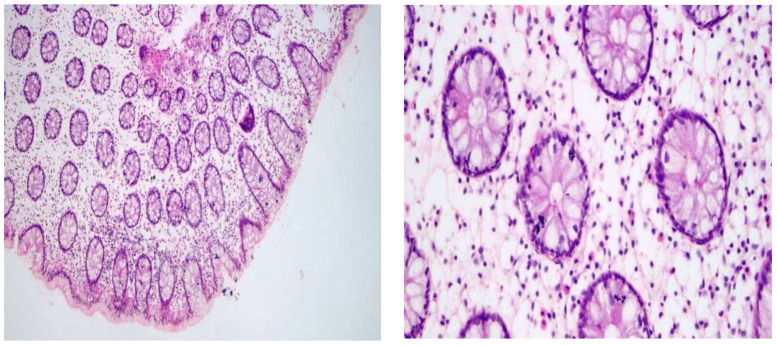
Mild focal colitis (quiescent stage of IBD)/(known case of ulcerative colitis).

**Figure 4:**
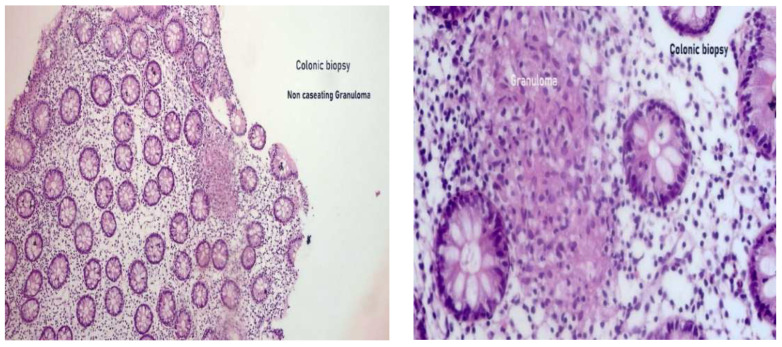
Moderate diffuse colitis with non-caseating epithelioid granulomatous inflammatory lesion highly suggestive of Crohn’s disease.

At the time of enrolment, 22 patients (27.5%) were receiving anti-TNF (anti-TNF) therapy, 39 patients (48.75%) were receiving (5-ASA), and 19 patients (23.75%) were drug-free. Among the treated patients, 59 were still in the active phase, and 21 showed endoscopically as being in remission. Clinical response for all patients was performed using the A MAYO partial score and Crohn’s Disease Activity Index (CDAI) score (Figure 1, Figure 2, Figure 3 and Figure 4).

Multiple snips of colonic mucosa. Lamina was widened by dense mixed inflammatory cellular infiltrate involving muscularis mucosa. The infiltrate was mainly lymphoplasmacytic with mixed neutrophils and eosinophils. There was crypt irregularity with branching and a reduction in mucin production. There was detected cryptitis and crypt abscesses (Left: 200×/Right: 400×, H&E staining).

Multiple snips of colonic mucosa with focal ulceration. Lamina was widened by dense mixed inflammatory cellular infiltrate involving muscularis mucosa. The infiltrate was mainly lymphoplasmacytic with mixed neutrophils and eosinophils. There was crypt irregularity with branching and a reduction in mucin production. Cryptitis and crypt abscesses were detected (Left: 200×/Right: 400×, H&E staining).

Multiple snips of colonic mucosa with preserved mucin production and no significant crypt irregularity. Lamina propria was the seat of chronic inflammatory cellular infiltrate with some eosinophils about 15/HPF. No detected cryptitis or crypt abscesses (Left: 200×/Right: 400×).

Snips of colonic mucosa. Crypts were regular. The lamina propria was the seat of moderate inflammatory cellular infiltrate. The infiltrate was formed of lymphocytes and neutrophils with focal cryptitis. Multiple epithelioid granulomas were detected, which were non-caseating (Left: 200×/Right: 400×, H&E staining).

### 2.2. Patients’ and Non-IBD Control Participants’ Characteristics

The IBD patients’ age, gender distribution, and smoking habits did not differ from those of the non-IBD control subjects. When patients were taken into account, there was no discernible difference in the length of the treatments for the 5-ASA and anti-TNF groups (Table 1).

### 2.3. Gene Expression among Studied Groups

**Figure 5:**
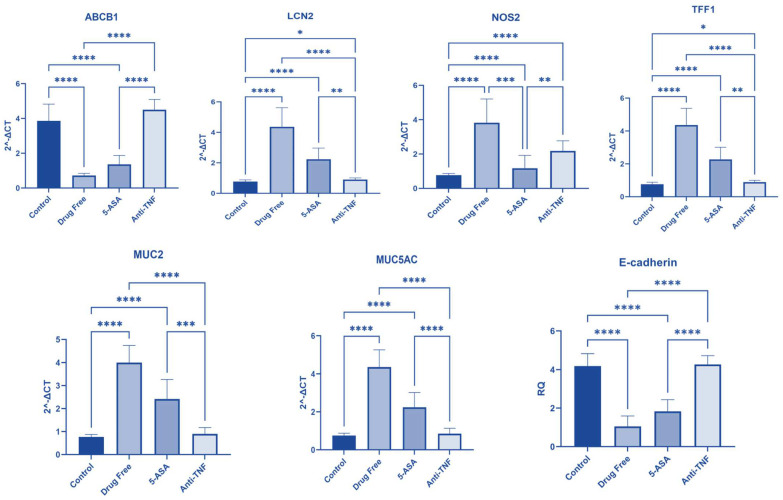
Gene expression in different groups. This shows the gene expression of various genes among the other studied groups (* significant at *p* < 0.05, ** at *p* < 0.01, *** at *p* < 0.001, **** at *p* < 0.0001).

The results are displayed in Table S1 and Figure 5.

#### 2.3.1. ABCB1 Gene Expression

Regarding ABCB1 gene expression, there was a statistically substantial difference between patients receiving anti-TNF-α and those receiving 5-ASA (*p* < 0.0001). There was also a significant difference between participants receiving anti-TNF-α and those who received no treatment (*p* < 0.0001). There was no statistical difference between patients obtaining anti-TNF-α and the control group (*p* = 0.4361).

#### 2.3.2. LCN2 Gene Expression

Regarding LCN2 gene expression, there was a statistically marked difference between participants receiving anti-TNF-α and those receiving 5-ASA (*p* = 0.0012). There was a statistically substantial difference between patients receiving anti-TNF-α and those who received no treatment (*p* < 0.0001). There was a statistically marked difference between patients obtaining anti-TNF-α and the control group (*p* = 0.0139).

#### 2.3.3. NOS2 Gene Expression

There was a statistically significant difference between participants receiving anti-TNF-α and those receiving 5-ASA in NOS2 gene expression (*p* = 0.0095). There was no statistically significant difference between patients receiving anti-TNF-α and those receiving no treatment (*p* > 0.9999). There was a statistically significant difference between the anti-TNF-α treated patients and the control group (*p* < 0.0001).

#### 2.3.4. TFF1 Gene Expression

Regarding TFF1 gene expression, patients receiving anti-TNF-α and those receiving 5-ASA differed statistically significantly (*p* = 0.0016). There was a statistically significant difference between the patients taking anti-TNF-α and those who received no treatment (*p* < 0.0001). There was a statistically marked difference between patients taking anti-TNF-α and the control group (*p* = 0.0188) [16].

#### 2.3.5. MUC2 Gene Expression

Regarding MUC2 gene expression, there was a statistically substantial difference between patients receiving anti-TNF-α and those receiving 5-ASA (*p* = 0.0003). There was a statistically significant difference between patients receiving anti-TNF-α and those who received no treatment (*p* < 0.0001). There was no statistically significant difference between patients receiving anti-TNF-α and the control group (*p* = 0.2564).

#### 2.3.6. MUC5AC Gene Expression

There was a statistically marked variation in MUC5AC gene expression between patients receiving anti-TNF-α and those receiving 5-ASA (*p* < 0.0001). There was a statistically marked difference between the patients receiving anti-TNF-α and those who received no treatment (*p* < 0.0001). There was no statistically significant difference between patients receiving anti-TNF-α and the control group (*p* > 0.9999).

#### 2.3.7. E-Cadherin Gene Expression

Regarding E-cadherin gene expression, there was a statistically substantial variation between those receiving anti-TNF-α and 5-ASA (*p* < 0.0001). There was a statistically marked difference between patients obtaining anti-TNF-α and those who received no treatment (*p* < 0.0001). There was no statistically substantial difference between patients taking anti-TNF-α and the control group (*p* > 0.9999) [17].

### 2.4. ROC Curve Analysis for Different Gene Expression

**Figure 6:**
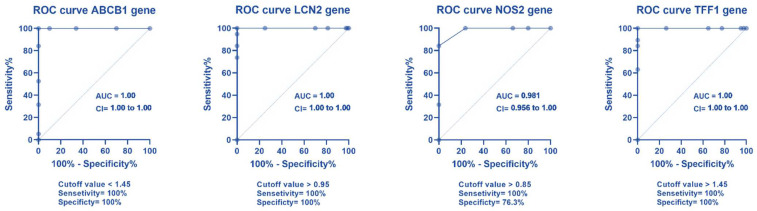

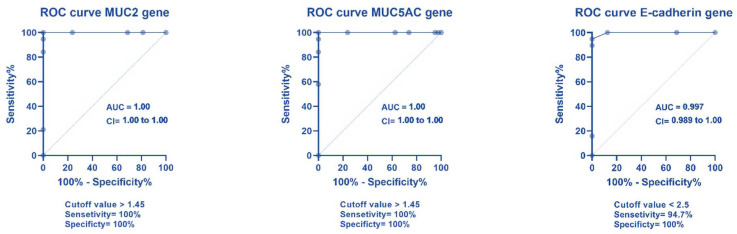
The ROC curve analysis of the different genes.

The results are displayed in Figure 6.

For discrimination between the IBD patients and controls, ROC curve analysis was performed for different genes. The ABCB1 gene’s cutoff value was <1.45, with a sensitivity of 100%, a specificity of 100%, and an AUC of 1. The AUC was 1, a sensitivity oof 100%, specificity was 100%, and >0.95 was the cutoff value for the LCN2 gene. The NOS2 gene’s cutoff value was >0.85, with a sensitivity of 100%, a specificity of 76.3%, and an AUC of 0.981. The TFF1 gene’s cutoff value was >1.45, with a sensitivity of 100%, a specificity of 100%, and an AUC of 1. The MUC2 gene’s cutoff value was >1.45, with a sensitivity of 100%, a specificity of 100%, and an AUC of 1. The MUC5AC gene’s cutoff value was >1.45, with a sensitivity of 100%, a specificity of 100%, and an AUC of 1. The E-cadherin gene’s cutoff value was <2.5, with a sensitivity of 94.7%, a specificity of 100%, and an AUC of 0.0997.

### 2.5. Correlation between Different Genes

**Figure 7:**
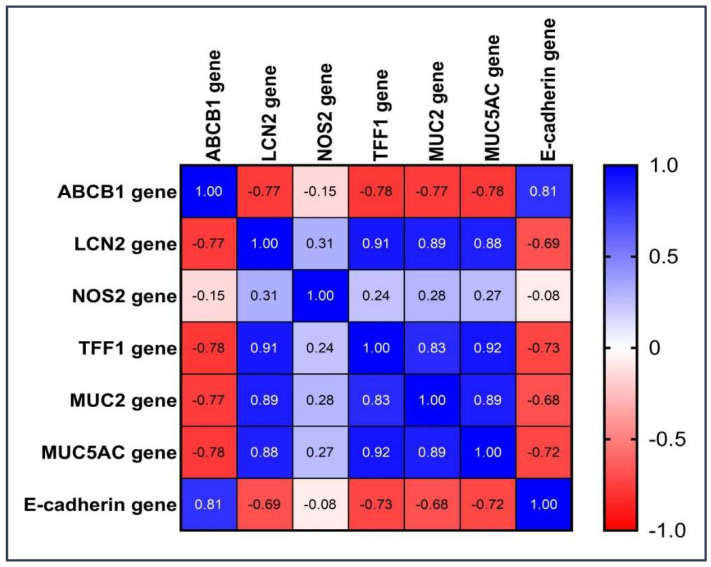
Correlation between different genes.

The correlation between different genes is explained in Figure 7 and Table S2. There was a strong negative relationship between the ABCB1 gene and E-cadherin gene, LCN2 gene, MUC2 gene, MUC5AC gene, and TFF1 gene (r = −0.73, −0.77, −0.76, −0.77, and −078 respectively). There was a negligible relationship between the ABCB1 gene and the NOS2 gene (r = −0.14).

There was a strong positive relationship between the LCN2 gene and E-cadherin gene, MUC2 gene, MUC5AC, and TFF1 gene (r = 0.88, 0.88, 0.87, 0.91, respectively). On the other hand, there was a moderate positive relationship between the LCN2 gene and the NOS2 gene (r = 0.31). There was a strong positive relationship between the MUC2 gene and both the E-cadherin gene and the MUC5AC gene (r = 0.9 and 0.88, respectively).

There was a strong relationship between the MU5AC gene and E-cadherin (r = 0.88).

There was a weak positive relationship between the NOS2 gene, MUC2 gene, MUC5AC gene, and TFF1 gene (r = 0.28, 0.26, and 0.23, respectively). On the other hand, there was a moderate positive relationship between the NOS2 gene and E-cadherin (r = 0.3).

There was a strong positive relationship between the TFF1 gene and the E-cadherin gene, MUC2 gene, and MUC5AC gene (r = 0.88, 0.83 and 0.91, respectively).

## 3. Discussion

In order to assess changes in the colonic mucosa of IBD patients under various therapy modalities, we used a bull’s eye gene expression approach to look at genes implicated in inflammation, apoptosis, immunological response, cellular adhesion, and tissue remodeling. Previous studies comparing the molecular signature of patients undergoing various medications remain underrepresented, despite the extensive use of the transcriptional analysis of intestinal tissues comparing IBD patients and non-IBD controls to uncover new biomarkers.

Following anti-TNF medication, ABCB1 was increased after being downregulated in drug-free patients. P-Glycoprotein 1, an ATP-dependent transmembrane pump that transfers drugs from the intracellular to the extracellular area, is encoded by the ABCB1 gene, commonly known as the MDR1 gene. It has been proposed that reduced gut expression levels and ABCB1′s lack of function are factors in the genesis of IBD. The typical gastrointestinal system expresses ABCB1 extensively [18].

Additionally, inconsistent results have been found in several genetic studies examining the connection between IBD susceptibility and the three SNPs (G2677T/A, C3435T, and C1236T) regarded to be the most clinically significant [19]. Numerous research has evaluated ABCB1′s role in the responsiveness to anti-TNF medications. There was no significant correlation between the ABCB1 gene alternates and infliximab response in Italian and Hungarian patients with IBD [11,20,21]. In this study, we found that the low levels of ABCB1 in patients were markedly increased by anti-TNF medication, bringing them to levels that were equivalent to the non-IBD controls. One in vitro study using Caco-2 cell lines displayed that in vitro TNF reduced the MDR1 mRNA levels. However, the possible alteration of ABCB1 in the mucosa of IBD patients by anti-TNF therapy has not been studied [22].

Additionally, we found that ABCB1 expression was significantly increased by anti-TNF medication, reaching levels comparable to those seen in the control subjects free of IBD. These results were consistent with the earlier research by Milanesi et al. (2019) [14].

Our findings demonstrate that 5-ASA and anti-TNF medications can lower the mucosal expression levels of NOS2, TFF1, and LCN2, which were excessive in untreated cases relative to the controls. Interestingly, anti-TNF medicine had a more significant effect on TFF1 and LCN2; nonetheless, treatment returned their levels to normal for individuals who were not affected. The impacts of 5-ASA on NOS2 expression also seem more favorable, despite the differences from the non-IBD controls being noticeably different. These results were in line with those of an earlier investigation by Milanesi et al. (2019) [14]. The NOS2 and LCN2 genes encode two antimicrobial peptides (AMPs): nitric oxide synthase 2 and the neutrophil gelatinase-associated lipocalin (NGAL) system.

LCN2/NGAL prevents bacterial development by securing iron-containing siderophores, while NOS2 produces nitric oxide, a reactive free radical that is a biological moderator in antibacterial, neurotransmission, and anti-tumor effects.

Numerous studies have demonstrated that the mucosa of IBD patients has both highly elevated LCN2/NGAL and NOS2 levels [23,24]. Notably, the NGAL protein has been proposed as a potential biomarker in IBD patients since, if present in high concentrations, it is positively connected with the severity and activity of the disease. The LCN2/NGAL levels were shown to be higher in patients with active UC, and infliximab treatment resulted in a decrease in these levels [24].

A single high infliximab dosage effectively lowered the elevated LCN protein concentration in the urine of CD patients. This intriguing result was also shown in our research, where the treated group’s LCN2 expression was lower [25].

Nitric oxide (NO) is produced by the enzyme NOS2, activated through a concoction of lipopolysaccharide and specific inflammatory mediators. Nitric oxide controls bowel epithelial cells, preserves the barrier’s integrity, and supports tight junctions. Additionally, it starts the oxidation of proteins and lipids through a free radical process that causes a redox imbalance. It has been demonstrated that the colon mucosa of both UC and CD patients exhibit enhanced nitric oxide synthase activity [26,27].

Higher gene expression levels were found by Senhaji et al. in 2017 in the colonic mucosa of CD patients compared to the controls and in patients with active UC compared to patients with passive UC [28]. Numerous populations have demonstrated a relationship between NOS2 gene variations and IBD risk [28,29]. Luther and colleagues discovered that the colonic expression of NOS2 was higher in non-responders than responders in individuals who lost response to the TNF-antagonist [30].

TFF1 is another increased gene in untreated individuals, and 5-ASA and anti-TNF treatments impact it. It is related to the same family as the trefoils TFF1, TFF2, and TFF3 [31]. It has been demonstrated that these persistent secretory proteins contribute to the upkeep and restoration of epithelial surfaces because they are highly expressed in the gastrointestinal mucosa [32].

While chronic inflammation encourages TFF expression to limit the course of the disease, acute mucosal damage stimulates TFFs to expedite cell migration to seal the wounded zone from luminal contents. TFF1 was expressed in individuals with severe UC in immunohistochemical studies on colon biopsy specimens but not in normal tissue [33].

Shaoul et al. (2004) found that this trefoil protein was also expressed in the colons of patients with IBD [34]. The serum levels of the trefoil factors have also been investigated, and those with IBD had higher concentrations of TFF1 and TFF3 [35].

Additionally, in UC patients, the serum TFF3 concentration was associated with the clinical and biochemical indicators of disease activity [36].

In the inflamed IBD colon, E-cadherin gene expression was dramatically reduced. The loss and destruction of epithelial cells in the colon affected by IBD are likely to blame for the reduced expression.

Additionally, IBD responders to anti-TNF medication showed normalized expression of this gene, and these results were consistent with those of an earlier study conducted by Arijs et al. in 2011 [37].

Both the MUC2 and MUC5AC genes were found to be significantly expressed in the current study’s untreated IBD patients, and their expression returned to normal after receiving anti-TNF medication.

MUC2 is the prominent colonic mucin in IBD, although it is not just found in healthy goblet cells. Clamp et al. [38] discovered that granular MUC2 is expressed by not phenotypical goblet cells in IBD and other inflammatory conditions of the colon. Contrary to the goblet cells of healthy people and IBD patients, who keep mature granular mucin and do not express immature mucin outside the Golgi, these cells express weakly glycosylated mucin present in secretory granules. This mucin is likely secreted as a juvenile, sparsely glycosylated product. This abnormal pattern of mucin glycosylation in IBD patients has also been proposed by others [38,39].

To make up for the loss of barrier and repair function caused by MUC2 and perhaps ITF expression alterations during inflammation, MUC5AC and TFF1 expression may be upregulated. It has been hypothesized that TFF1 aids in healing and regeneration [40].

Goblet cells that express MUC5ACTFF1 are frequently found in inflammatory conditions, indicating that this is a generic response to inflammation and may not always signify dysplastic alterations [34]. The cross-sectional nature of this study could be a possible drawback. Furthermore, the small size of our study prevented us from conducting additional subgroup analysis (UC and CD separately).

## 4. Materials and Methods

### 4.1. Patients

**Figure 8:**
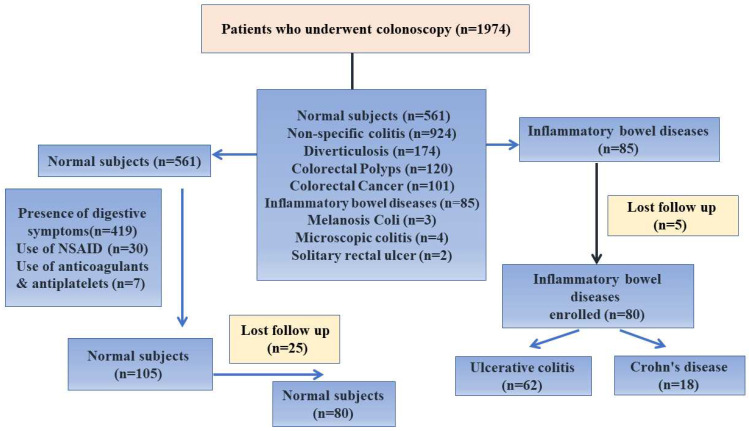
Flowchart of the study.

Eighty IBD patients and 80 healthy control participants from the Tropical Medicine Department at the Mansoura Faculty of Medicine were recruited for this study out of 1974 patients who underwent colonoscopy over three years (Figure 8).

Multiple colonoscopic biopsies were taken from the inflamed mucosa in patients with active IBD, those IBD patients who were in remission, and from the healthy colonic mucosa from healthy control individuals. Before biopsy sampling, all individuals provided written informed consent. The Mansoura Faculty of Medicine’s Institutional Review Board (MFM-IRB) authorized the study (Code number R.22.08.1786).

Crohn’s disease (CD) and ulcerative colitis (UC) were identified in accordance with suggestions made by the European Crohn’s and Colitis Organization (ECCO), which preclude that the diagnosis of CD or UC is based on a combination of clinical, biochemical, stool, endoscopic, cross-sectional imaging, and histological investigations [3]. According to Dobre et al. (2018) [14], the following exclusion criteria were applied to the non-IBD control subjects: the presence of gastrointestinal symptoms, current or previous use of non-steroidal anti-inflammatory drugs (within the last three months), and current or prior use of anticoagulants/antiplatelet drugs (within the last three months).

Monoclonal antibodies directed against TNF-α are fast-acting and potent anti-inflammatory agents. Anti-TNF therapies approved for treating IBD include infliximab, adalimumab, and certolizumab [3].

### 4.2. Total RNA Isolation and qPCR

For qPCR, a 7500 Fast Real-Time PCR system was used. The total RNA was extracted from fresh-frozen tissues using the RNeasy Mini Kit (Qiagen, Venlo, Germany), and the RNA quantity and quality were assessed using a spectrophotometric approach (NanoDrop 2000, Thermo Scientific™ ND2000USCAN, Waltham, MA, USA). The total RNA was then converted to complementary DNA (cDNA) using the SensiFASTTM cDNA Kit (Bioline, London, UK). As an internal control gene, glyceraldehyde 3-phosphate dehydrogenase (GAPDH) was employed along with the SensiFASTTM SYBR Green PCR Master Mix from Bioline. We applied the 2CT approach to determine the fold change in gene expression. Table S3 shows the primer sequences of different genes, and the GAPDH gene was used as the control gene.

### 4.3. Statistical Analysis

Continuous variables were tested using the t-test, while categorical variables were tested using the Chi-square test. The gene expression data are displayed as the mean ± standard deviation (SD). To ascertain each gene’s appropriate cutoff value and diagnostic accuracy, receiver operating characteristic (ROC) curves were built.

The differences in gene expression were estimated using Kruskal–Wallis and Dunn’s multiple comparisons tests. The statistical analysis of each gene’s expression was verified for normality using the Shapiro–Wilk normality test. The Spearman correlation test was used to examine the relationship among genes. GraphPad Prism was used for statistical analysis (version 9.3.1). At *p* < 0.05, significance was accepted.

## 5. Conclusions

Considering the potential drawbacks of our investigation, we identified a configuration of genes whose ectopic expression in IBD mucosa appears to be better controlled by biological therapy (anti-TNF therapy) than by 5-ASA medications. Lipocalin-2 (LCN2), Nitric Oxide Synthase 2 (NOS2), Mucin 2 (MUC2), Mucin 5AC (MUC5AC), and Trefoil factor 1 (TFF1) were overexpressed in the untreated IBD patients compared to the non-IBD patients and that 5ASA and anti-TNF-a treatment reduced these expressions. Anti-TNF therapy helped the levels of ABCB1 and E-cadherin in the untreated IBD patients return to normal. To find the gene expression profiles useful for assisting therapeutic decision-making, more extensive studies of treatment naive IBD with standardized sampling and the prospective follow-up of clinical outcomes are pertinent.

## Figures and Tables

**Table 1:** Characteristics of the IBD and control participants.

IBD Patients	N = 80
Age (mean ± SD)	46.75 ± 6.648
Sex (%M)	(N = 56) 70%
Sex (%F)	(N = 24) 30%
Smoking Behavior	
Non-smokers	(N = 62) 77.5%
Mild	(N = 12) 15%
Moderate	(N = 3) 3.75%
Severe	(N = 3) 3.75%
Type of Disease	
(%UC)	(N = 62) 77.5%
(%CD)	(N = 18) 22.5%
Disease Activity	
% Active	(N = 59) 73.75%
% Remission	(N = 21) 26.25%
Treatment Duration	
Drug-free	23.75% (19 patients)
5-ASA treatment	48.75% (3–24 months) (31 UC patients & 8 CD patients)
Anti-TNF treatment	27.5% (3–24 months) (12 UC patients & 10 CD patients)
Non-IBD subjects	N = 80
Age (mean ± SD)	49.78 ± 4.352
Sex (%M)	(N = 55) 68.75%
Sex (%F)	(N = 25) 31.25%
Smoking Behavior	
Non-smokers	(N = 70) 87.5%
Mild	(N = 5) 6.25%
Moderate	(N = 2) 2.5%
Severe	(N = 3) 3.75%

## Data Availability

The data presented in this study are available on request.

## Supplementary Materials

The following supporting information can be downloaded at: https://www.mdpi.com/article/10.3390/ph16020324/s1, Table S1. Gene expression analysis among different groups, Table S2. Explanation of correlation between different genes, Table S3. Primer sequences of different genes.
