# Supplementary material for: Mucosal Genes Expression in Inflammatory Bowel Disease Patients: New Insights
Source: Pharmaceuticals (Basel). 2023 Feb 20;16(2):324. doi: 10.3390/ph16020324 (PMC9966817; doi:10.3390/ph16020324)

Table S1. Gene expression analysis among different groups

	$2^{-\Delta Ct}$ values					
**Gene**	**Control**	**Drug Free**	**5-ASA**	**Anti-TNF**	***p* value**	
**ABCB1**	3.86 ± 0.96	0.73 ± 0.12	1.37 ± 0.51	4.50 ± 0.60	Control vs. Drug Free	<0.0001
					Control vs. 5-ASA	<0.0001
					Control vs. Anti-TNF	0.4361
					Drug Free vs. 5-ASA	0.1312
					Drug Free vs. Anti-TNF	<0.0001
					5-ASA vs. Anti-TNF	<0.0001
**LCN2**	0.77 ± 0.11	4.37 ± 1.26	2.23 ± 0.74	0.90 ± 0.11	Control vs. Drug Free	<0.0001
					Control vs. 5-ASA	<0.0001
					Control vs. Anti-TNF	0.0139
					Drug Free vs. 5-ASA	0.4428
					Drug Free vs. Anti-TNF	<0.0001
					5-ASA vs. Anti-TNF	0.0012
**NOS2**	0.77 ± 0.10	3.83 ± 1.38	1.17 ± 0.75	2.18 ± 0.59	Control vs. Drug Free	<0.0001
					Control vs. 5-ASA	<0.0001
					Control vs. Anti-TNF	<0.0001
					Drug Free vs. 5-ASA	0.0004
					Drug Free vs. Anti-TNF	>0.9999
					5-ASA vs. Anti-TNF	0.0095
**TFF1**	0.76 ± 0.13	4.37 ± 1.01	2.28 ± 0.73	0.90 ± 0.10	Control vs. Drug Free	<0.0001
					Control vs. 5-ASA	<0.0001
					Control vs. Anti-TNF	0.0188
					Drug Free vs. 5-ASA	0.2639
					Drug Free vs. Anti-TNF	<0.0001
					5-ASA vs. Anti-TNF	0.0016
**MUC2**	0.77 ± 0.10	4.00 ± 0.75	2.42 ± 0.84	0.90 ± 0.27	Control vs. Drug Free	<0.0001
					Control vs. 5-ASA	<0.0001
					Control vs. Anti-TNF	0.2564
					Drug Free vs. 5-ASA	0.1811
					Drug Free vs. Anti-TNF	<0.0001
					5-ASA vs. Anti-TNF	0.0003
**MUC5AC**	0.75 ± 0.13	4.37 ± 0.90	2.24 ± 0.77	0.86 ± 0.28	Control vs. Drug Free	<0.0001
					Control vs. 5-ASA	<0.0001
					Control vs. Anti-TNF	>0.9999
					Drug Free vs. 5-ASA	0.1717
					Drug Free vs. Anti-TNF	<0.0001
					5-ASA vs. Anti-TNF	<0.0001
**E-cadherin**	4.19 ± 0.64	1.05 ± 0.54	1.83 ± 0.62	4.27 ± 0.46	Control vs. Drug Free	<0.0001
					Control vs. 5-ASA	<0.0001
					Control vs. Anti-TNF	>0.9999
					Drug Free vs. 5-ASA	0.4972
					Drug Free vs. Anti-TNF	<0.0001
					5-ASA vs. Anti-TNF	<0.0001

Table S2. Explanation of correlation between different genes

r value =	
**+.70 or higher**	Very strong positive relationship
**+.40 to +.69**	Strong positive relationship
**+.30 to +.39**	Moderate positive relationship
**+.20 to +.29**	weak positive relationship
**+.01 to +.19**	No or negligible relationship
**0**	No relationship [zero correlation]
**-.01 to -.19**	No or negligible relationship
**-.20 to -.29**	weak negative relationship
**-.30 to -.39**	Moderate negative relationship
**-.40 to -.69**	Strong negative relationship
**-.70 or higher**	Very strong negative relationship

Table S3. Primer Sequences of different genes

Gene	Primer Sequences	Reference Sequences	PCR product size (bp)
GAPDH	**F:** ATGTTGCAACCGGGAAGGAA<br>**R:** AGGAAAAGCATCACCCGGAG	NM_001256799.3	159bp
**ABCB1 gene**	**F:**TCAGGAAGCAACCAGATAAAAGAG<br>**R:**CAATCAGCCTCACCACAGATG	NM_000927.5	141bp
**LCN2 gene**	**F:**GTTACCTCGTCCGAGTGGTG<br>**R:**CCGAAGTCAGCTCCTTGGTT	XM_047423376.1	126bp
**NOS2 gene**	**F:**CGCATGACCTTGGTGTTTGG<br>**R:**CATAGACCTTGGGCTTGCCA	NM_000625.4	142bp
**TFF1 gene**	**F:**ATACCATCGACGTCCCTCCA<br>**R:**TGGGACTAATCACCGTGCTG	NM_003225.3	104bp
**MUC2 gene**	**F:**AGTTTGGGGAGCACTTCGAG<br>**R:**TCTTCCACGCAGTGGGTAAC	>NM_002457.4	118BP
**MUC5AC gene**	**F:**CGACCTGTGCTGTGTACCAT<br>**R:**GTGCAGGGTCACATTCCTCA	NM_001304359.2	197BP
**E-cadherin gene**	**F:**TGGACCGAGAGAGTTTCCCT<br>**R:**TTAGCCTCGTTCTCAGGCAC	NM_004360.5	172BP

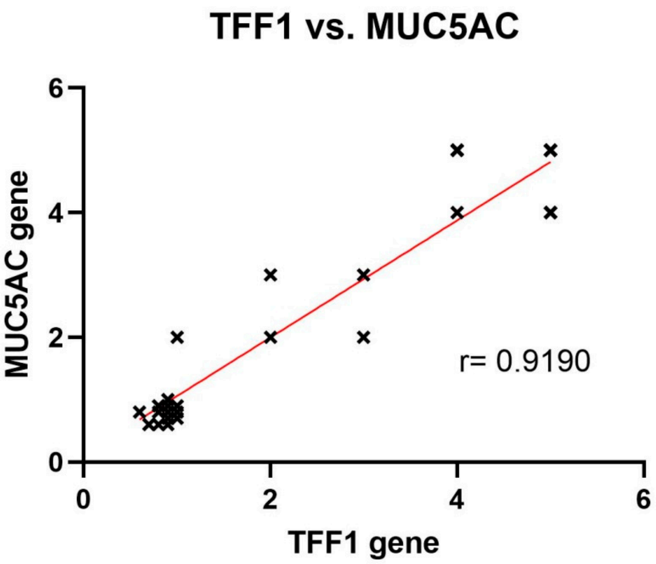
TFF1 vs. MUC5AC
MUC5AC gene
TFF1 gene
r= 0.9190
0
2
4
6

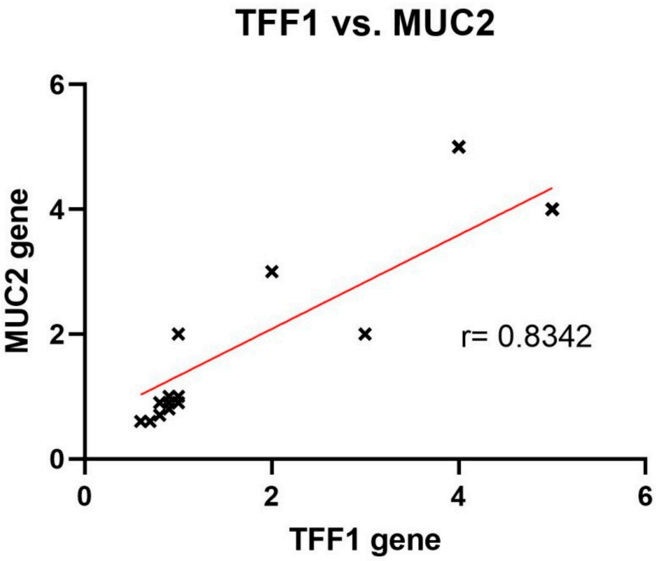
TFF1 vs. MUC2
MUC2 gene
TFF1 gene
r= 0.8342
0
2
4
6

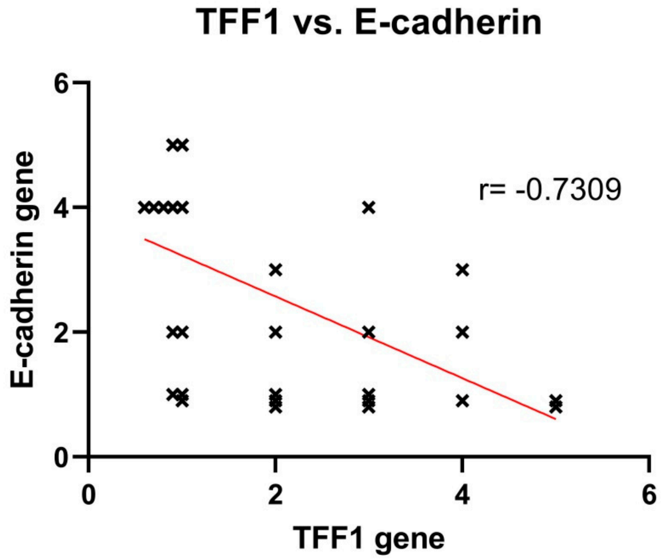
TFF1 vs. E-cadherin
r= -0.7309
E-cadherin gene
TFF1 gene
0
2
4
6

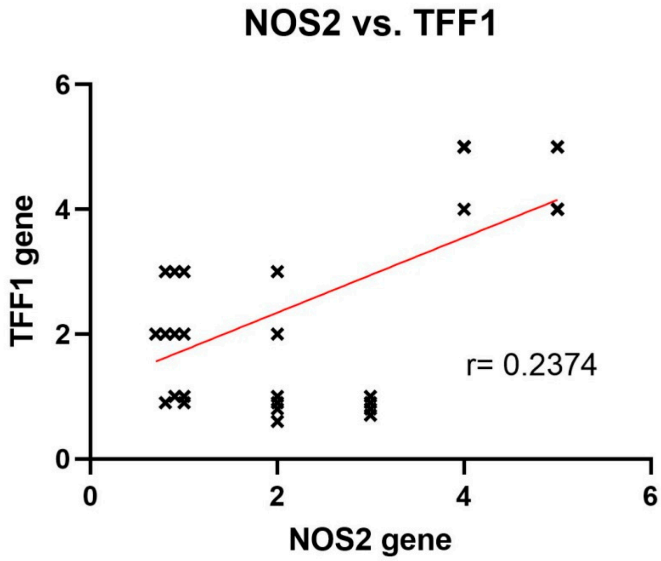
NOS2 vs. TFF1
r= 0.2374
TFF1 gene
NOS2 gene
0
2
4
6

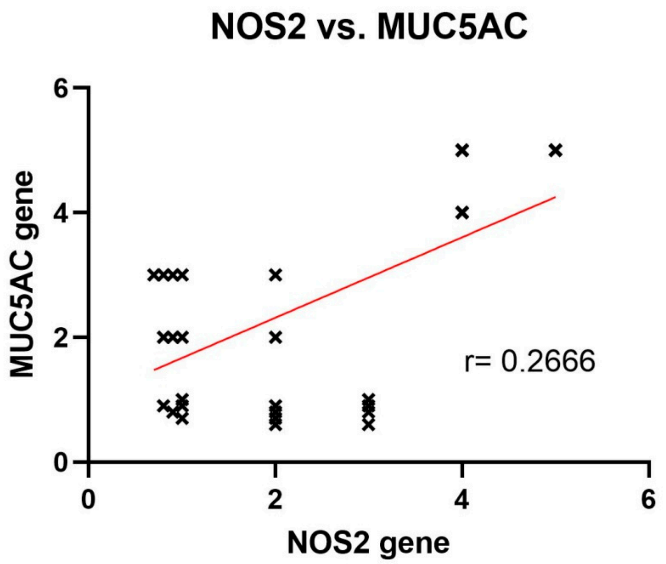
NOS2 vs. MUC5AC
MUC5AC gene
NOS2 gene
r= 0.2666
0
2
4
6

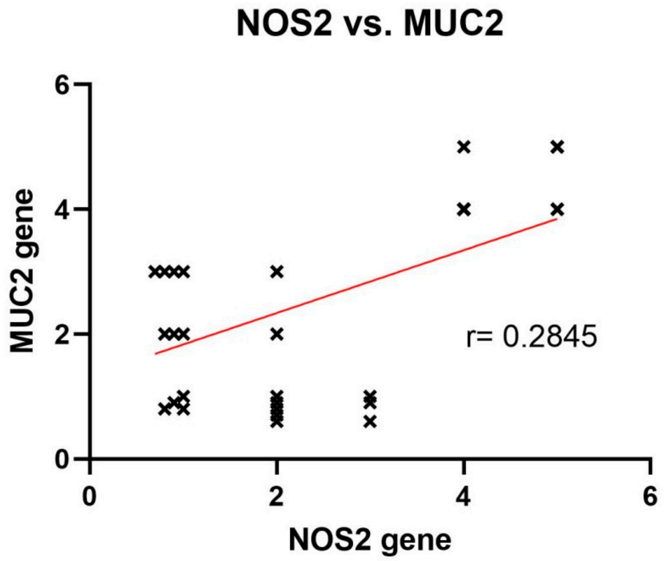
NOS2 vs. MUC2
MUC2 gene
NOS2 gene
r= 0.2845
0
2
4
6

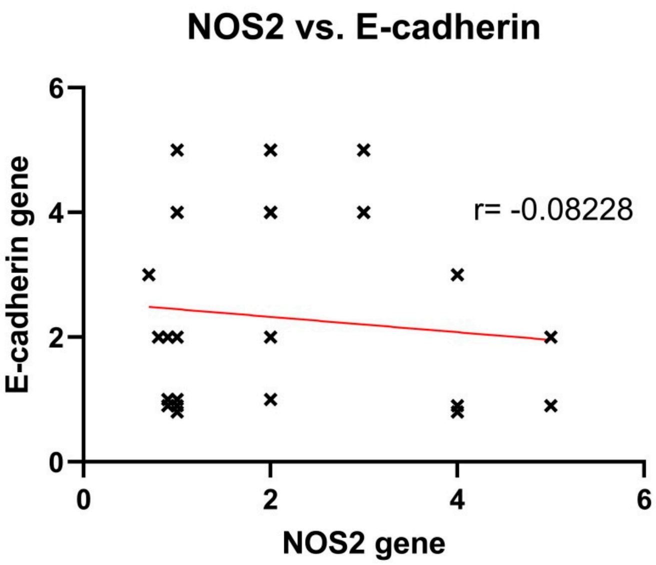
NOS2 vs. E-cadherin
r= -0.08228
E-cadherin gene
NOS2 gene
0
2
4
6

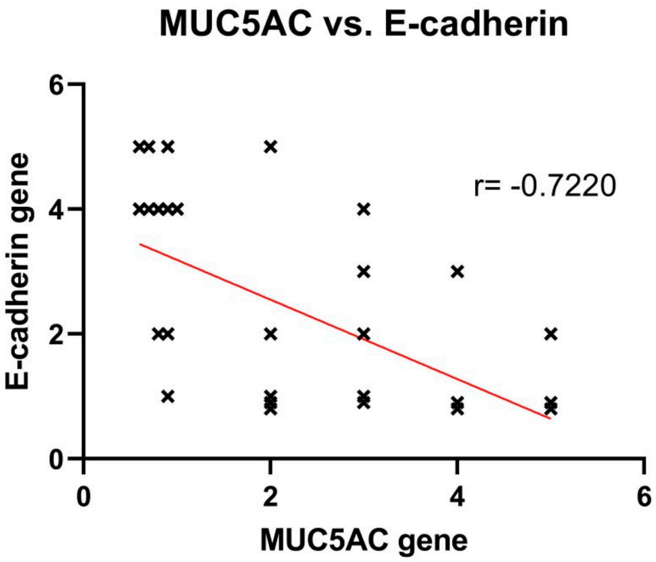
MUC5AC vs. E-cadherin
r= -0.7220
E-cadherin gene
MUC5AC gene
0
2
4
6

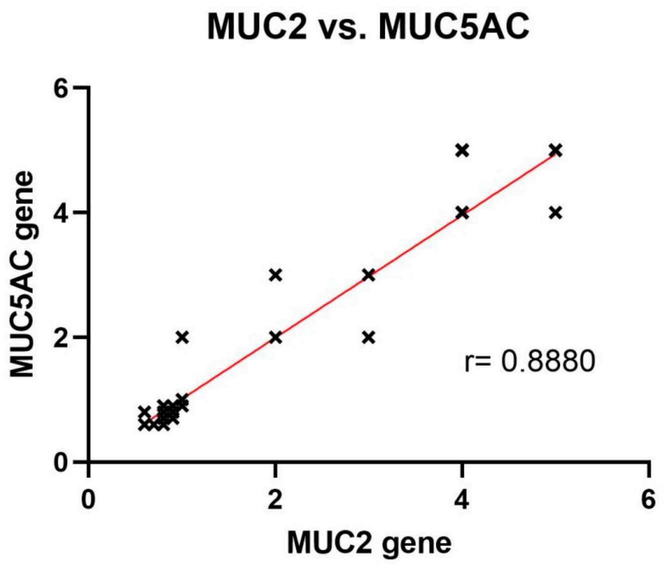

MUC2 vs. MUC5AC
MUC5AC gene
MUC2 gene
r= 0.8880
0
2
4
6

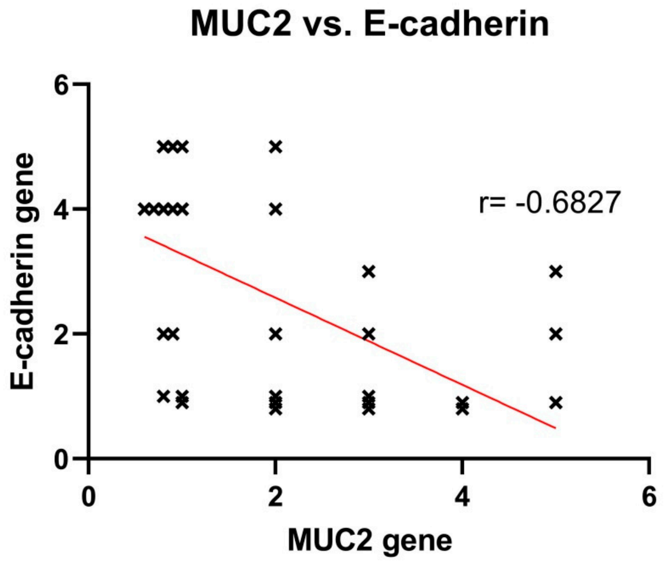

MUC2 vs. E-cadherin
E-cadherin gene
MUC2 gene
r= -0.6827
0
2
4
6

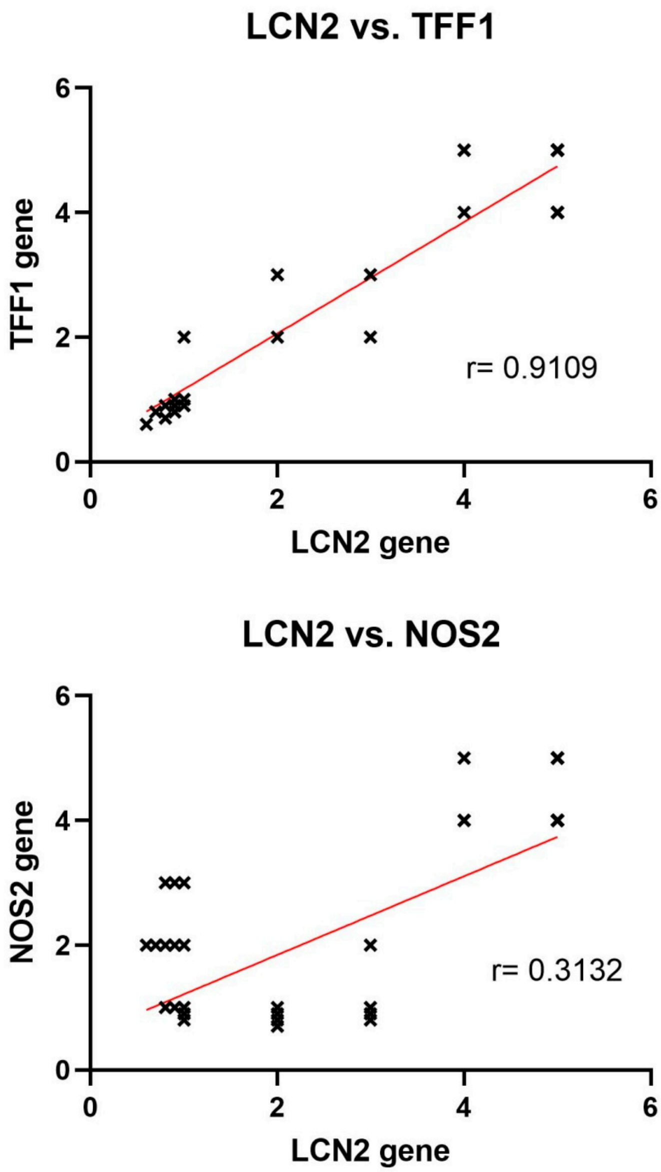
LCN2 vs. TFF1
TFF1 gene
LCN2 gene
r= 0.9109
0
2
4
6
LCN2 vs. NOS2
NOS2 gene
LCN2 gene
r= 0.3132

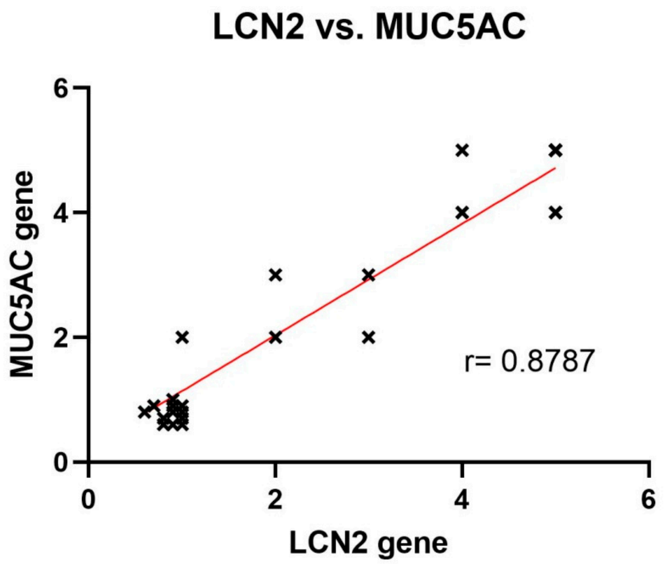

LCN2 vs. MUC5AC
MUC5AC gene
LCN2 gene
r= 0.8787
0
2
4
6

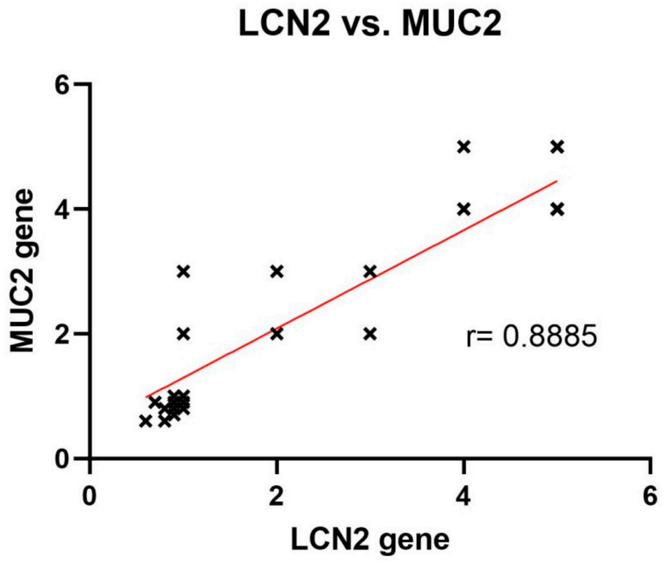

LCN2 vs. MUC2
MUC2 gene
LCN2 gene
r= 0.8885
0
2
4
6

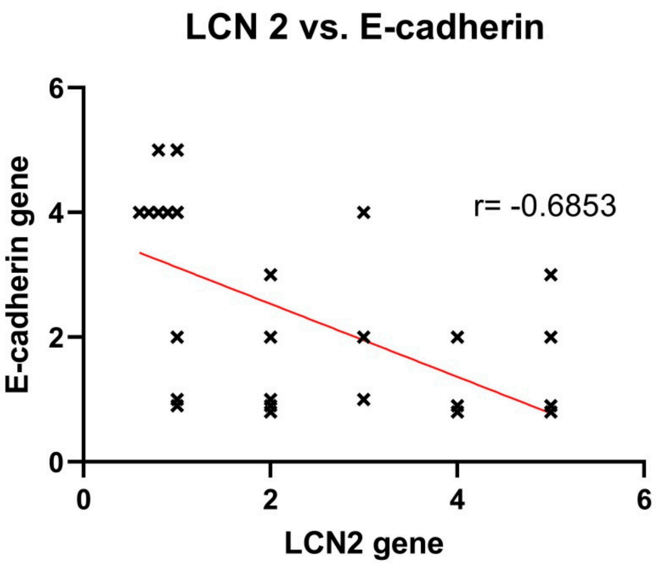
LCN 2 vs. E-cadherin
E-cadherin gene
LCN2 gene
r= -0.6853
0
2
4
6

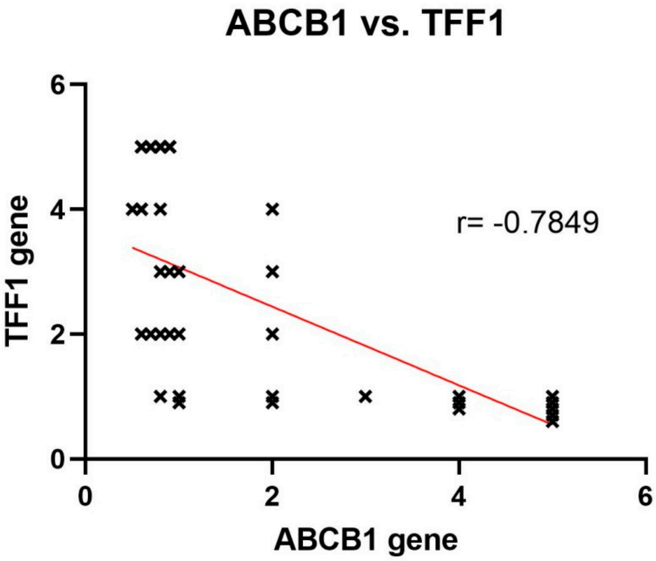
ABCB1 vs. TFF1
TFF1 gene
ABCB1 gene
r= -0.7849
0
2
4
6

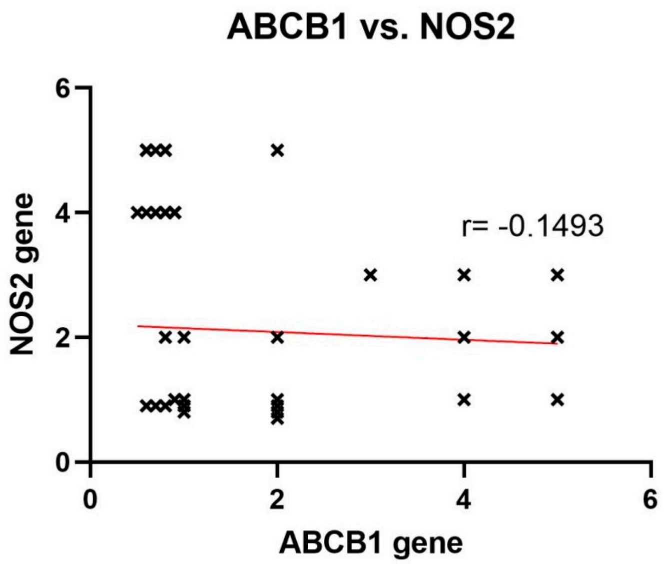
ABCB1 vs. NOS2
r= -0.1493
NOS2 gene
ABCB1 gene
0
2
4
6

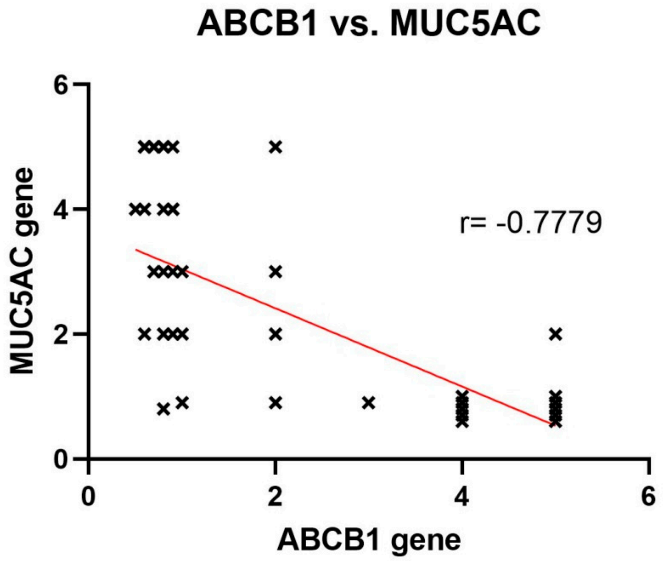
ABCB1 vs. MUC5AC
r= -0.7779
MUC5AC gene
ABCB1 gene
0
2
4
6

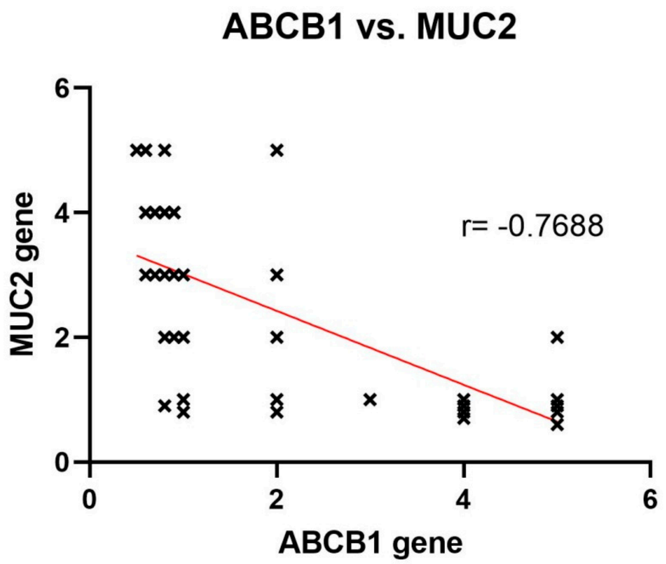
ABCB1 vs. MUC2
MUC2 gene
ABCB1 gene
r= -0.7688
0
2
4
6

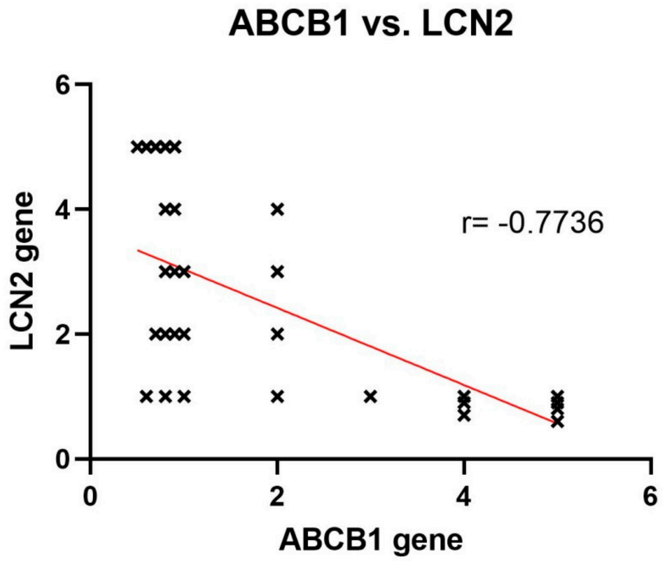
ABCB1 vs. LCN2
LCN2 gene
ABCB1 gene
r= -0.7736
0
2
4
6

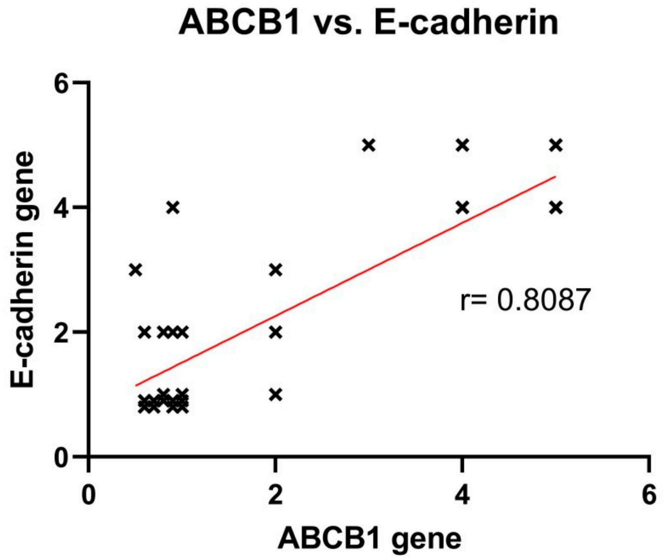
ABCB1 vs. E-cadherin
E-cadherin gene
ABCB1 gene
r= 0.8087
0
2
4
6
0
2
4
6

IBD Patients

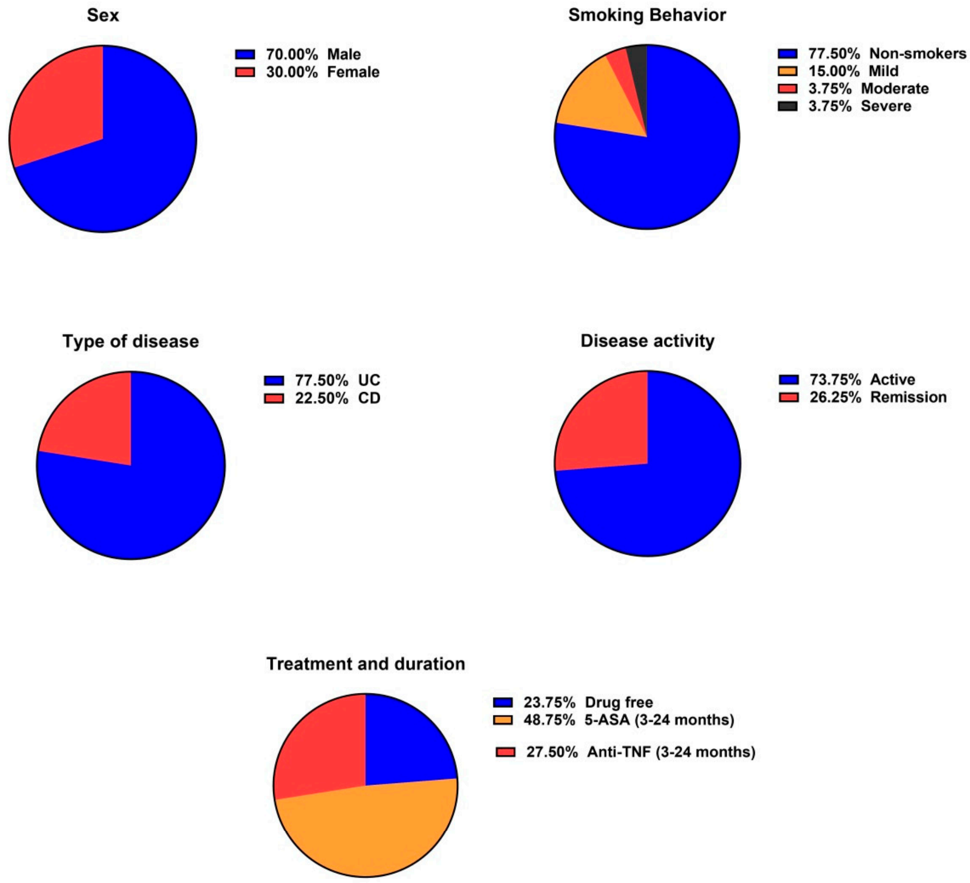

Non-IBD controls

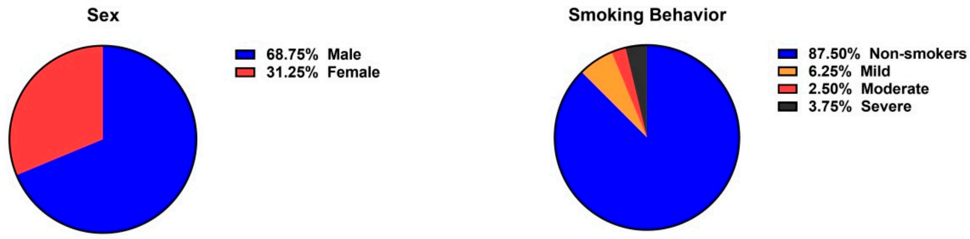

Supplement: Supplementary file 1 [file pharmaceuticals-16-00324-s001.zip › pharmaceuticals-2188407-supplementary.pdf]
